# Can selenium supplementation modify oxidative stress *in-vitro*? A role for selenium supplementation in the prevention of cardiovascular disease

**DOI:** 10.1186/1476-9255-12-S1-P7

**Published:** 2015-04-16

**Authors:** Dean Leighton, Marie Goua, Eimear Dolan, Katherine Burgess, Giovanna Bermano

**Affiliations:** 1 Institute for Health and Wellbeing Research, Robert Gordon University, Aberdeen, AB10 7GJ, UK

## Background

Two thirds of the UK population are either overweight or obese (body mass index (BMI) 25-29.9 and >30 kg/m^2^ respectively) and are typically characterised by systemic oxidative stress (OS); deemed to play a key role in cardiovascular disease (CVD) development. OS results from chronically high reactive oxidative species (ROS) formation and reduced antioxidant status. OS plays a key role in CVD development by initiating atherosclerosis (fatty plaque accumulation within the arterial walls); therefore obese individuals are at increased risk of atherosclerosis development. Increased monocyte ROS generation instigates atherosclerotic plaque formation by increasing the recruitment, binding and transmigration of monocytes across arterial endothelial cells and into the arterial wall. An increased dietary antioxidant intake or up-regulation of endogenous antioxidant enzymes may counteract this OS state and therefore lower CVD risk. Selenium is an essential dietary micronutrient incorporated within the catalytic site of endogenous antioxidant Glutathione Peroxidase (GPx) enzymes, which protect cells from OS and consequent cell damage. There is, however, a lack of knowledge concerning the effect of selenium supplementation in an OS state representative of sedentary overweight/obese individuals. The aim of this work was to investigate the ability of selenium supplementation to modify monocyte cell viability/ROS production under OS.

## Materials and methods

U937 monocyte cells were supplemented with sodium selenite (Na_2_SeO_3_; 100nM or 200nM) or not and cultured for 48 hours at 37°C. Paraquat (100mM) and S-Nitroso-N-acetyl-DL-penicillamine (10mM) (PQ/SNAP) were added to the cells to induce OS. Cell viability was assessed *via* MTS (3-(4,5-dimethylthiazol-2-yl)-5-(3-carboxymethoxyphenyl)-2-(4-sulfophenyl)-2H-tetrazolium) assay while ROS production was determined by Flow Cytometry using the reagent CM-H_2_DC-FDA.

## Results

PQ/SNAP treatment significantly reduced U937 cell viability and significantly increased ROS generation compared to untreated control U937 cells (p<0.001); confirming the induction of OS. U937 cells cultured in medium containing either 100nM or 200nM Na_2_SeO_3_ were significantly less affected by OS. More specifically, 100nM Na_2_SeO_3_ supplementation significantly improved cell viability by 33% (p<0.001) and significantly attenuated ROS generation by 32% (p<0.001) when compared to un-supplemented PQ/SNAP treated cells. Additionally, 100nM Na_2_SeO_3_ supplementation resulted in a significantly greater increase in cell viability compared to 200nM Na_2_SeO_3_ supplementation of PQ/SNAP treated cells (p<0.01).

## Conclusions

This study indicates that selenium supplementation may be effective in reducing the detrimental effects of OS by significantly improving cell viability and ROS quenching ability; thus providing preliminary data to support the potential role for selenium supplementation with regards to CVD prevention of overweight/obese individuals.

